# Profiles of a broad spectrum of epigenetic DNA modifications in normal and malignant human cell lines: Proliferation rate is not the major factor responsible for the 5-hydroxymethyl-2′-deoxycytidine level in cultured cancerous cell lines

**DOI:** 10.1371/journal.pone.0188856

**Published:** 2017-11-30

**Authors:** Marek Foksinski, Ewelina Zarakowska, Daniel Gackowski, Magdalena Skonieczna, Karolina Gajda, Dorota Hudy, Anna Szpila, Karol Bialkowski, Marta Starczak, Anna Labejszo, Jaroslaw Czyz, Joanna Rzeszowska-Wolny, Ryszard Olinski

**Affiliations:** 1 Department of Clinical Biochemistry, Faculty of Pharmacy, L. Rydygier Collegium Medicum in Bydgoszcz, Nicolaus Copernicus University in Toruń, Bydgoszcz, Poland; 2 Biosystems Group, Institute of Automatic Control, Faculty of Automatics, Electronics, and Informatics, Silesian University of Technology, Gliwice, Poland; 3 Biotechnology Centre, Silesian University of Technology, Gliwice, Poland; 4 Department of Hematology, Faculty of Medicine, L. Rydygier Collegium Medicum in Bydgoszcz, Nicolaus Copernicus University in Toruń, Bydgoszcz, Poland; University of Navarra, SPAIN

## Abstract

Active demethylation of 5-methylcytosine moiety in DNA occurs by its sequential oxidation to 5-hydroxymethylcytosine, 5-formylcytosine and 5-carboxycytosine, catalysed by enzymes of the Ten-Eleven Translocation family proteins (TETs 1, 2 and 3). Here we analyzed for the first time all the intermediate products of DNA demethylation pathway in the form of deoxynucleosides (5-methyl-2′-deoxycytidine, 5-(hydroxymethyl)-2′-deoxycytidine, 5-formyl-2′-deoxycytidine and 5-carboxy-2′-deoxycytidine as well as 5-(hydroxymethyl)-2′-deoxyuridine) using automated isotope-dilution online two-dimensional ultra-performance liquid chromatography with tandem mass spectrometry. DNA was isolated from human malignant cell lines of colon adenocarcinoma (HCT 116), melanoma (Me45), myelogenous leukemia bone marrow blasts (K562), EBV-positive Burkitt’s lymphoma lymphoblasts (Raji), EBV-negative Burkitt’s lymphoma lymphoblasts (male-CA46 and female-ST486), as well as normal neonatal dermal fibroblasts (NHDF-Neo). The expression levels of *TET1*, *TET2*, *TET3*, *SMUG1*, and *TDG* genes were also assayed by RT-qPCR. Our results show a global erasure of 5-hydroxymethyl-2′-deoxycytidine and 5-carboxy-2′-deoxycytidine in DNA of cultured cells compared with DNA from primary malignant tissue. Moreover, malignant cells in culture have a quite different DNA epigenetic profile than cultured normal cells, and different types of malignant cells display different and characteristic profiles of DNA epigenetic marks. Similar analyses of a broader spectrum of epigenetic modifications, not restricted to 5-methyl-2′-deoxycytidine, could lead to better understanding of the mechanism(s) responsible for emergence of different types of cancer cells.

## Introduction

DNA methylation is involved in diverse biological processes including gene expression, which in turn has a profound impact on cellular identity and organismal fate [1]. It was recently found that cytosine methylation in cellular DNA is much more dynamic than previously appreciated. Active DNA demethylation is carried out by the Ten-Eleven Translocation enzymes (TET 1, 2 and 3), which catalyze oxidation of 5-methyl-2′-deoxycytidine (5-mdC) moiety in DNA to form 5-(hydroxymethyl)-2′-deoxycytidine (5-hmdC) and further oxidation reactions that generate 5-formyl-2′-deoxycytidine (5-fdC) and 5-carboxy-2′-deoxycytidine (5-cadC) (reviewed in [1, 2]). 5-FdC and 5-cadC are subsequently recognized and removed by thymine-DNA glycosylase (TDG) in base excision repair process. Some experimental evidence supports the hypothesis that 5-(hydroxymethyl)-2′-deoxyuridine (5-hmdU) may also be generated by TET enzymes and has epigenetic functions [3].

Tissue type is a major modifier of the 5-hmdC level and there is a substantial, about 10-fold, reduction of this level in cell lines when compared with their tissue of origin [4, 5]. Moreover, this reduction is linked to rapid reprogramming of epigenetic and transcriptional states [5], but nothing is known as to whether the other TET products 5-fdC and 5-cadC follow the same rule. There have been no studies concerning differences in epigenetic DNA modifications between normal and malignant cell lines or differences between malignant cell lines.

Since downstream steps of active demethylation processes resulting in products of iterative oxidation of (5-fdC, 5-cadC) may be at least partially responsible for the loss of 5-hmdC, in this study we investigated all described to date TETs products in the form of 2′-deoxynucleosides generated by enzymatic hydrolysis of DNA samples, i.e. 5-mdC, 5-hmdC, 5-fdC and 5-cadC, as well as 5-hmdU. We used several malignant human cell lines: colon adenocarcinoma (HCT 116), malignant melanoma (Me45), myelogenous leukemia bone marrow blasts (K562), Burkitt’s lymphoma EBV-positive lymphoblasts (Raji), and Burkitt’s lymphoma EBV-negative lymphoblasts (male-CA46 and female-ST486, described in this publication as BL), as well as normal human cells, namely neonatal dermal fibroblasts (NHDF-Neo). In addition, we compared the pattern of demethylation products in tissue samples of human colorectal cancer (CRC) and cultured HCT 116 cells, as well as in leukocytes from acute myelogenous leukemia (AML) patients and K562 cell line.

DNA modifications were assayed using a recently-developed, isotope-dilution online two-dimensional ultra-performance liquid chromatography with tandem mass spectrometry (2D-UPLC-MS/MS) [6]. The level of transcripts of several genes involved in the formation of epigenetic DNA marks/modifications (*TETs 1–3*, *SMUG1*, *TDG*) was also determined.

## Materials and methods

### Cell cultures

NHDF-Neo, HCT 116, K562, Raji, BL (Ca46 and ST486) cells were obtained from ATCC. The Me45 cell line was derived in 1997 from a lymph node metastasis of skin melanoma of a 35 year-old male at the Radiobiology Department of the Centre of Oncology in Gliwice [7]. Cells were grown at 37°C in a humidified atmosphere with 5% CO_2_ using DMEM/F-12 medium (GE Medical Systems Polska) for HCT 116, Me45 and NHDF-Neo cells and RPMI (Sigma-Aldrich) for K562, Raji, Ca46 and ST486 cells, supplemented with 10% fetal bovine serum (Eurx) and penicillin-streptomycin (Sigma-Aldrich). Cell doubling times were determined by counting cells in a hemocytometer. Cells were harvested in the exponential phase of growth for DNA isolation.

### Study group

Patients included in this study represent a group of the 65 patients with surgically resected, colorectal cancer and 10 patients with confirmed AML diagnosis. All patients were recruited by the hospital of L. Rydygier Collegium Medicum in Bydgoszcz, Nicolaus Copernicus University in Toruń, Poland. The study was approved by the medical ethics committee of the L. Rydygier Collegium Medicum in Bydgoszcz, Nicolaus Copernicus University in Toruń, Poland (No. KB/400/2013 and KB/404/2016) and all the patients gave informed consent.

### Isolation of leukocytes from AML patients

Venous blood samples (18 mL) from the AML patients were collected. The blood was carefully applied on top of Histopaque 1119 solution (Sigma-Aldrich) and leukocytes were isolated by centrifugation according to the procedure supplied by the manufacturer.

### DNA extraction and hydrolysis to deoxynucleosides

Preparation of cell pellet from of CRC tissues was described previously [6]. The pellet of the cells and tissues was dispersed by vortexing in ice-cold buffer B [10 mM Tris-HCl, 5 mM Na_2_EDTA, 0.15 mM deferoxamine mesylate, pH 8.0 (Sigma-Aldrich)]. Solutions of RNase A (Sigma-Aldrich) and RNase T1 (Sigma-Aldrich) were added to the final concentrations of 0.5 mg/mL and 100 U/mL, respectively, and vortexed again. Sodium dodecyl sulfate (Sigma-Aldrich) solution was added to the final concentration of 0.5% m/v and the mixture was gently mixed using a polypropylene Pasteur pipette. After 30 min incubation at 37°C, proteinase K (Sigma-Aldrich) was added to the final concentration of 1 mg/mL. The mixture was incubated at 37°C for 1 h, cooled to 4°C, transferred to a centrifuge tube with 1 volume of phenol/chloroform/isoamyl alcohol mixture [25:24:1 (v/v/v)] (Sigma-Aldrich) and vortexed vigorously. The aqueous phase separated by centrifugation was vortexed with a 1 volume of chloroform/isoamyl alcohol mixture [24:1 (v/v)] (J.T.Baker) and centrifuged again. Supernatant containing DNA was treated with two volumes of cold absolute ethanol in order to precipitate high molecular weight DNA. The precipitate was removed with a plastic spatula and washed with 70% (v/v) ethanol. DNA (typically 30–100 μg) was dissolved in 100 μL of 100 mM ammonium acetate (Sigma-Aldrich) containing 0.2 mM ZnCl_2_ (Avantor Performance Materials Polska S.A.), pH 4.3. The dissolved DNA sample was mixed with the solutions containing 1 U of nuclease P1 (Sigma-Aldrich), 10 μg tetrahydrouridine (Calbiochem) as cytidine deaminase inhibitor, and incubated at 37°C for 1 h. Subsequently, 12 μL of 10% (v/v) NH_4_OH (J.T.Baker) and 1.3 U of alkaline phosphatase (Sigma-Aldrich) were added to the mixture followed by 1 h incubation at 37°C. Finally, DNA hydrolysate was ultrafiltered using cut-off 10 kDa multi-well plate and concentrated by evaporation in SpeedVac down to 20 μL system for approximately 40 min.

### 2D-UPLC–MS/MS analysis

The analyses were performed using a method described earlier [6]. Briefly, DNA hydrolysates were spiked with a mixture of internal standards in volumetric ratio 4:1, to concentration of 50 fmol/μL of [D_3_]-5-hmdC, [^13^C_10_, ^15^N2]-5-fdC, [^13^C_10_, ^15^N_2_]-5-cadC, and [^13^C_10_, ^15^N_2_]-5-hmdU. Chromatographic separation was performed with a Waters Acquity 2D-UPLC system with photo-diode array detector for the first dimension chromatography (used for quantification of unmodified deoxynucleosides and 5-mdC) and Xevo TQ-S tandem quadrupole mass spectrometer for second dimension chromatography. At-column-dilution technique was used between first and second dimension for improving retention at trap/transfer column. The columns used were: a Phenomenex Kinetex C-18 column (150 mm×2.1 mm, 1.7 μm) at the first dimension, a Waters X-select C18 CSH (100 mm×2.1 mm, 1.7 μm) at the second dimension and Waters X-select C18 CSH (30 mm×2.1 mm, 1,7 μm) as trap/transfer column. Chromatographic system operated in heart-cutting mode, indicating that selected parts of effluent from the first dimension were directed to trap/transfer column *via* 6-port valve switching, which served as “injector” for the second dimension chromatography system. The flow rate at the first dimension was 0.25 mL/min and the injection volume was 0.5–2 μL. The separation was performed with a gradient elution for 10 min using a mobile phase 0.1% acetate (A) and acetonitrile (B) (1–5% B for 5 min, column washing with 30% acetonitrile and re-equilibration with 99% A for 3.6 min). Flow rate at the second dimension was 0.35 mL/min The separation was performed with a gradient elution for 10 min using a mobile phase 0.01% acetate (A) and methanol (B) (4–50% B for 4 min, isocratic flow of 50% B for 1.5 min, and re-equilibration with 96% A up to next injection). All samples were analyzed in three to five technical replicates of which technical mean was used for further calculation. Mass spectrometric detection was performed using the Waters Xevo TQ-S tandem quadrupole mass spectrometer, equipped with an electrospray ionization source. Collision-induced dissociation was obtained using argon 6.0 at 3 x 10^−6^ bar pressure as the collision gas. Transition patterns for all the analyzed compounds, as well as specific detector settings were determined using the MassLynx 4.1 Intelli-Start feature.

### Reverse transcription and real-time quantitative polymerase chain reaction (RT-qPCR)

RNA was extracted with Total RNA mini kits (A&A Biotechnology) and reverse transcribed using NG dART kits (Eurx) using oligo(dT). RT-qPCR was performed on a BioRad CFX 96 System using the Real-Time 2x PCR Master Mix SYBR A kit (A&A Biotechnology). Reaction mixtures were incubated for 2 min at 50°C, 4 min at 95°C, followed by 40 cycles of 45 sec at 95°C, and 30 sec at 55–60°C (depending on the primer set). Levels of expression were estimated by the ΔCT method [8, 9]. Sequences of primers for RT-qPCR were summarized in S1 Table.

### Statistical analysis

Statistical analysis was performed using STATISTICA version 12.5 (www.statsoft.com). Results are expressed as means±SD for variables with normal distribution for all cell lines, and as median with interquartile range for variables with nonparametric distribution.

Student’s t-test was performed for variables with normal distribution to assess the differences in the levels of epigenetic DNA modifications. Repeated-measures analysis of variance (ANOVA)–NIR test was performed to assess the differences in the levels of genes expression. The levels of transcripts of the genes *TET1*, *TET2*, *TET3*, *SMUG1* and *TDG* were performed in three biological repeats. For variables with nonparametric distribution Mann-Whitney’s U test was carried out. In all analyses p<0.05 was considered as statistically significant.

## Results

### Levels of cytosine and thymine modifications

**Fig 1:**
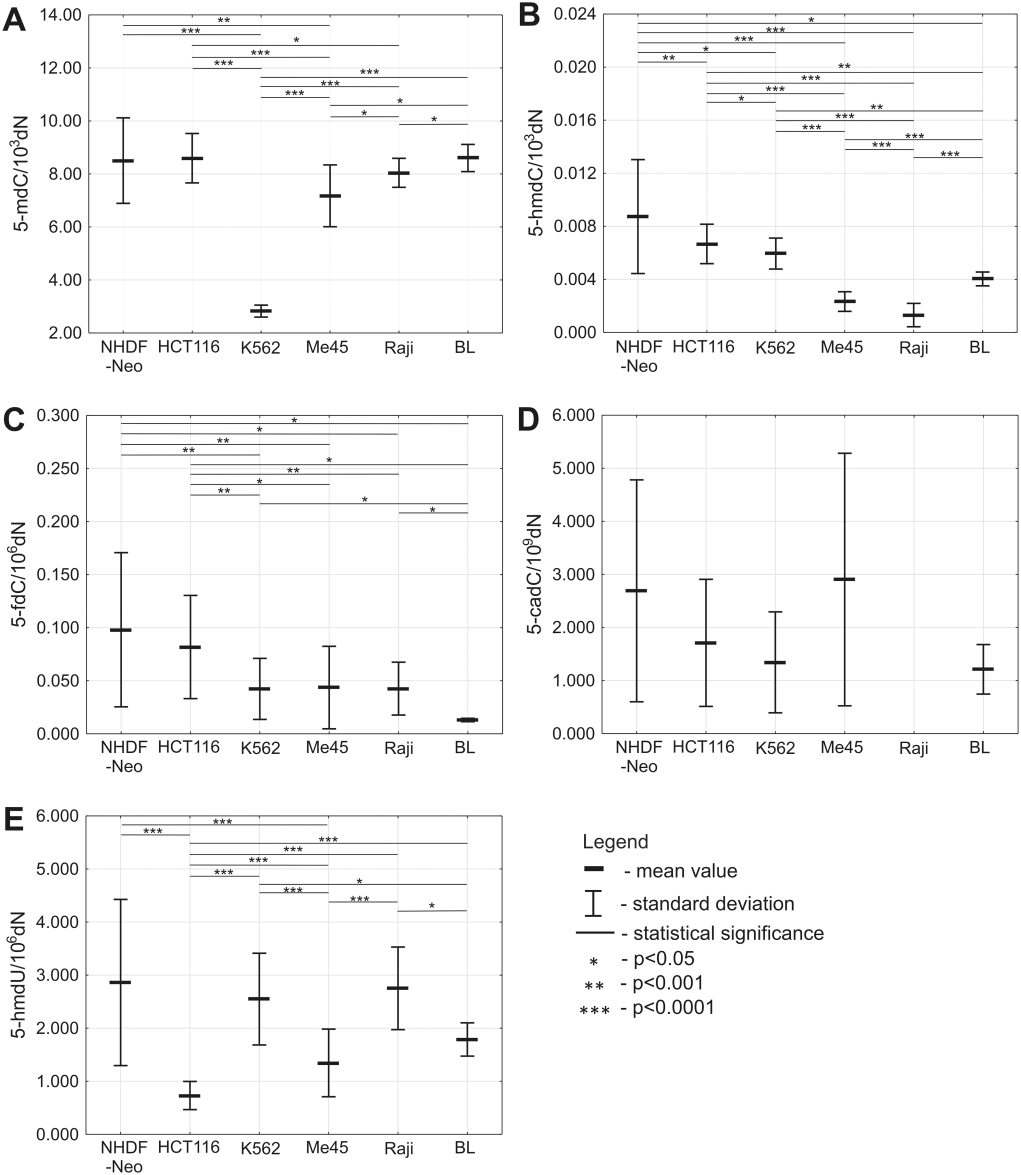
Levels of 5-mdC and intermediate products of active DNA demethylation in DNA from normal fibroblasts and various malignant cell lines. (A) Level of 5-mdC. (B) Level of 5-hmdC. (C) Level of 5-fdC. (D) Level of 5-cadC. (E) Level of 5-hmdU.

All currently identified products of 5-mdC and active DNA demethylation pathway were analyzed in DNA from normal (NHDF-Neo) and malignant (HCT 116, K562, Me45, Raji, BL) cell lines (Fig 1). The mean content of 5-mdC in DNA from NHDF-Neo cells was 8.50 (±SD 1.6130) per 10^3^ deoxynucleosides (dN). A significantly lower levels of 5-mdC were observed in K562 and Me45 cells with the mean values of 2.83/10^3^dN±0.2261 and 7.17/10^3^dN±1.1659, respectively. Also the 5-mdC level showed statistically difference in DNA from HCT 116 cell line (8.59/10^3^dN±0.9316) when compared to K562, Me45, Raji (mean value 8.04/10^3^dN±0.5471) cell lines, as well as in DNA from Me45 compared to Raji and BL (8.60/10^3^dN±0.5138) cell lines. Interestingly, in Burkitt’s lymphoma EBV-positive Raji cell line we observed significantly lower level of 5-mdC compared to the mean level calculated for EBV-negative cell lines BL (p = 0.0308) (Fig 1A).

The level of 5-hmdC in DNA from NHDF-Neo cells reached a mean value of 0.0087/10^3^dN±0.0043. We observed statistically significant lower level of this modification in DNA isolated from malignant cell lines with the following mean values: HCT 116 (0.0067/10^3^dN±0.0015), K562 (0.0059/10^3^dN±0.0012), Me45 (0.0023/10^3^dN±0.0007), Raji (0.0013/10^3^dN±0.0009), and BL (0.0040/10^3^dN±0.0005). Moreover, the level of this modification was very low in DNA from Me45 and Raji cell lines. We also observed that determination of 5-hmdC in DNA of Burkitt’s lymphoma EBV-negative cell lines BL revealed 3-fold higher level of this modification in comparison with EBV-positive Raji cell line (Fig 1B).

Similarly, higher level of 5-fdC was observed in normal NHDF-Neo cells (0.098/10^6^dN±0.0726) than in malignant HCT 116 (0.082/10^6^dN±0.0486), K562 (0.042/10^6^dN±0.0288), Me45 (0.044/10^6^dN±0.0389), Raji (0.043/10^6^dN±0.0249), as well as BL cell lines that demonstrated exceptionally low 0.013/10^6^dN±0.0016 5-fdC content in DNA (Fig 1C).

Interestingly, no significant differences in the level of 5-cadC were found for the all cell line pairs (Fig 1D). It should be mentioned that in most samples the level of this modification was at the limit of detection (several modifications per genome).

The highest level of 5-hmdU was observed in DNA from NHDF-Neo cells (2.86/10^6^dN±1.5655). We also observed statistically lower level of this modification in HCT 116 and Me45 cell lines, with respective mean values 0.73/10^6^dN±0.2657 and 1.35/10^6^dN±0.6373, as compared to normal NHDF-Neo cells. Moreover, statistically significant differences in 5-hmdU level were observed between HCT 116 and other malignant cell lines, with following mean values: K562 (2.55/10^6^dN±0.8643), Me45 (1.35/10^6^dN±0.6373), Raji (2.75/10^6^dN±0.7780), and BL (1.79/10^6^dN±0.3135) (Fig 1E).

**Fig 2:**
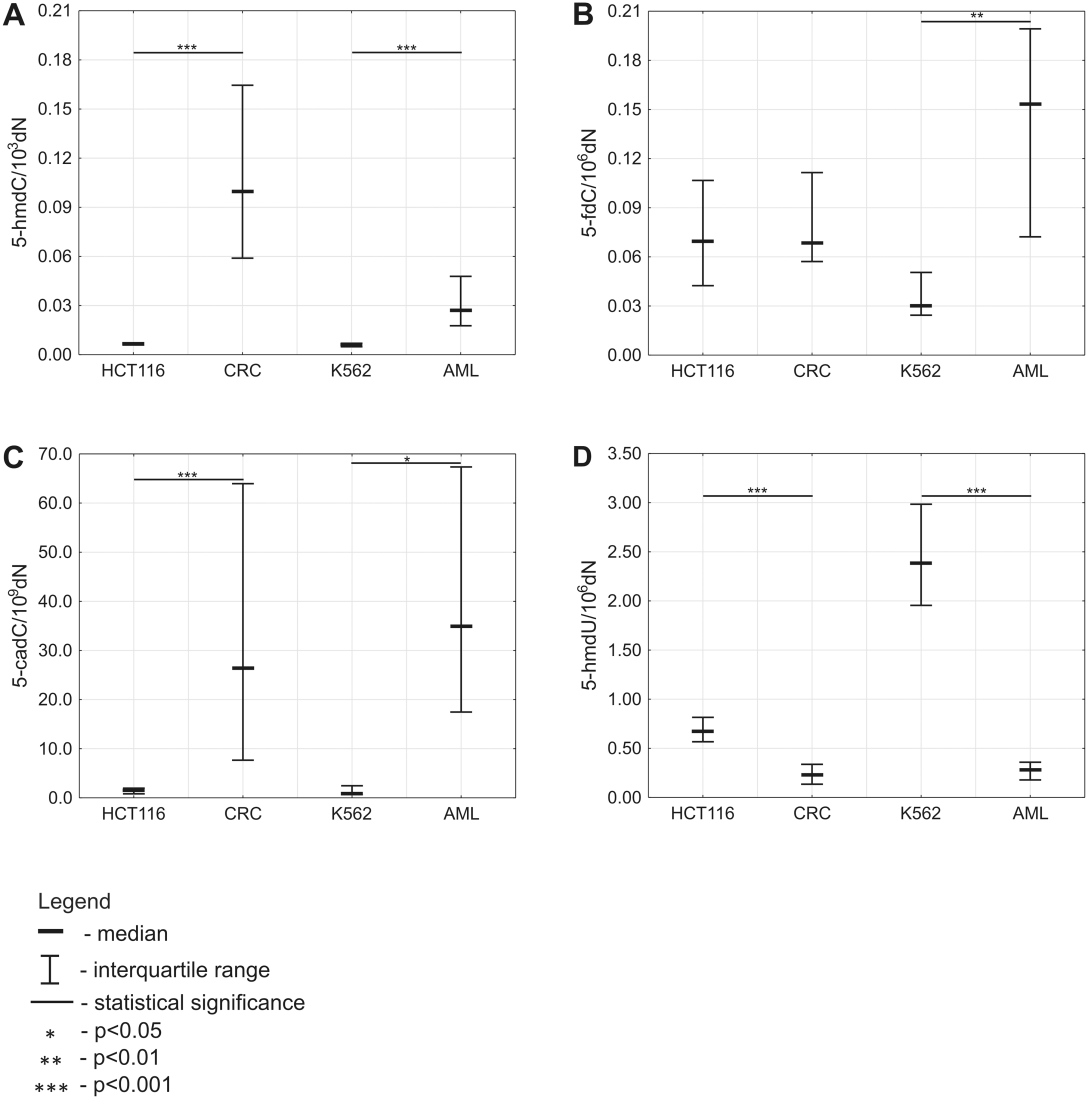
Levels of intermediate products of active DNA demethylation in DNA from cultured cells compared with DNA from primary malignant tissue. (A) Level of 5-hmdC. (B) Level of 5-fdC. (C) Level of 5-cadC. (D) Level of 5-hmdU.

In our study, the median of 5-hmdC and 5-cadC in DNA extracted from CRC tissues was 0.10/10^3^dN (0.0589–0.1645) and 26.40/10^9^dN (7.6614–63.9474), respectively. Thus, determination of these modifications in DNA from HCT 116 revealed respectively 15- and 16-fold lower level compared to CRC tissues (Fig 2A and 2C). In contrast, the level of 5-hmdU was higher in HCT 116 (0.67/10^6^dN (0.5668–0.8154)) compared to CRC tissues (0.23/10^6^dN (0.1350–0.3372)) (Fig 2D).

Determination of 5-hmdC in DNA from AML revealed significantly higher level of the modification in the blood isolated cells (0.03/10^3^dN (0.0177–0.0478)) compared to corresponding K562 cell line (0.01/10^3^dN (0.0048–0.0070)) (Fig 2A). We also observed statistically higher level of 5-cadC in leukocytes of AML patients (34.98/10^9^dN (17.4653–67.3516)) as compared to K562 cell line (0.87/10^9^dN (0.7000–2.4621)) (Fig 2C). On contrary, the level of 5-hmdU was 8.5-fold higher in K562 cell line (2.39/10^6^dN (1.9545–2.9840)) compared to primary tissue (0.28/10^6^dN (0.1783–0.3586)) (Fig 2D).

Interestingly, levels of 5-fdC were significantly higher (4.5-fold) in leukocytes of AML pastients compared to K562. No significant differences were observed between levels of this modification in DNA from HCT 116 cell line and DNA from CRC tissues (Fig 2B).

### The level of transcripts of several genes involved in the formation of epigenetic DNA marks/modifications (*TETs 1–3*, *SMUG1*, *TDG*)

**Fig 3:**
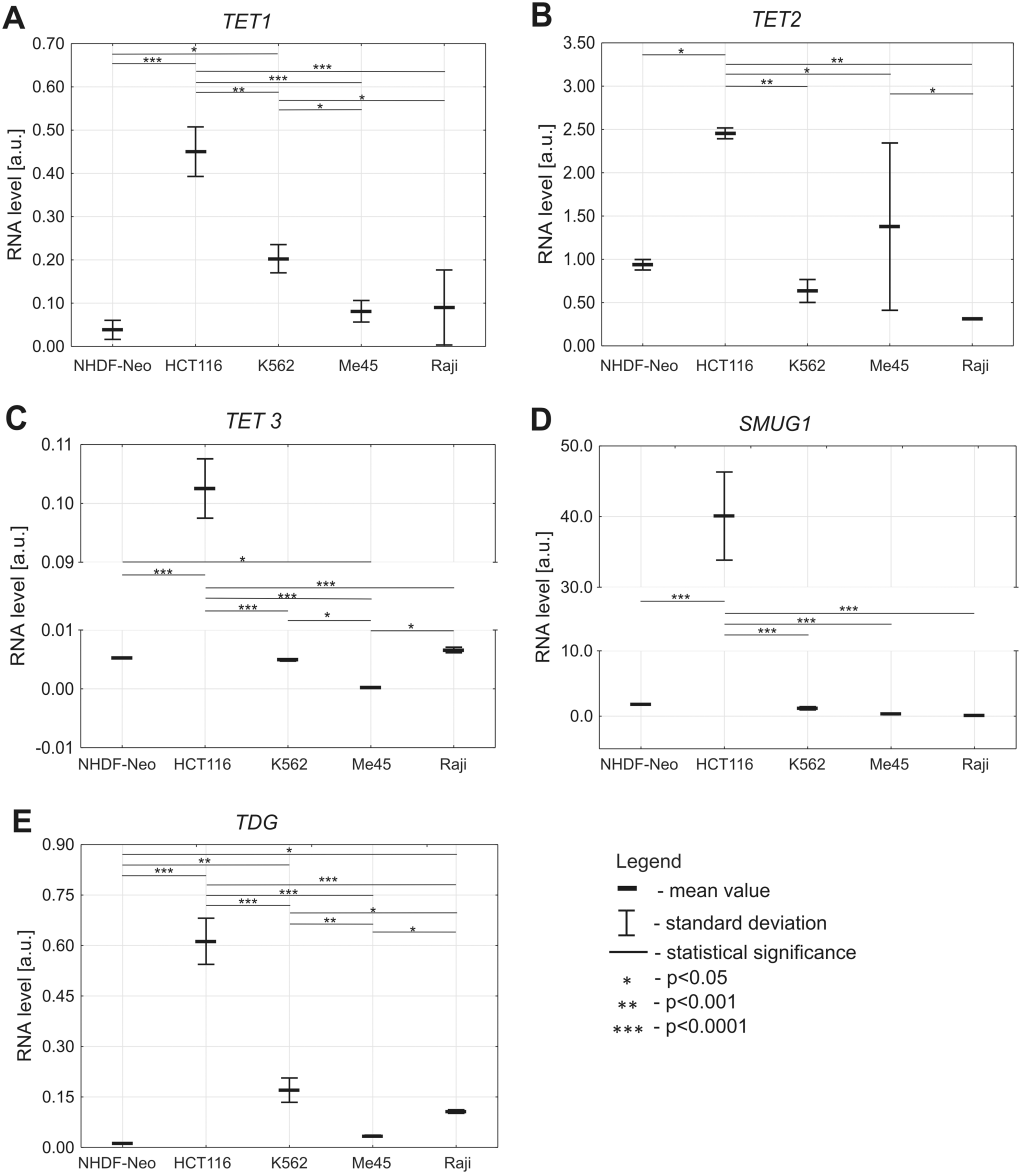
Levels of transcripts of the *TETs*, *SMUG1* and *TDG* genes in various cell lines. (A) *TET1*. (B) *TET2*. (C) *TET3*. (D) *SMUG1*. (E) *TDG*.

Using RT-qPCR with *GAPDH* as reference gene the levels of *TET1*, *TET2*, *TET3*, *SMUG1* and *TDG* transcripts were compared in NHDF-Neo, HCT 116, K562, Me45 and Raji cells (Fig 3). The mRNA levels of transcripts were calculated in relation to the *GAPDH*, a house keeping gene, and are presented as fold of the level of this gene expression.

**Fig 4:**
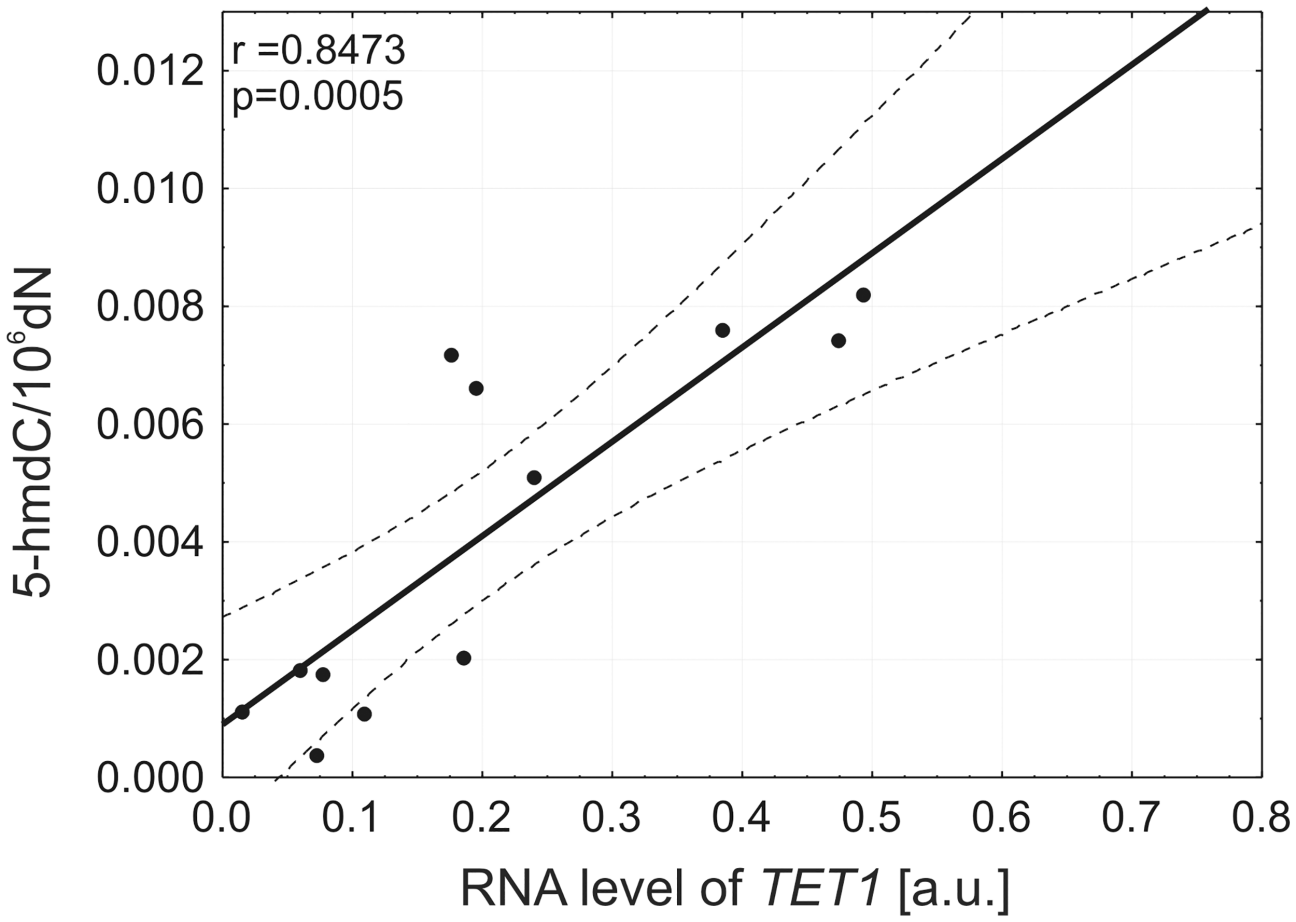
A positive correlation between 5-(hydroxymethyl)-2′-deoxycytidine and mRNA expression level of *TET1* gene in malignant cell lines.

We found a low amount of *TET1* transcript in NHDF-Neo cells. Moreover, we observed significantly higher levels of *TET1* transcript in HCT 116 (p<0.0001) and K562 (p = 0.0027) cell lines. Significantly lower amounts of *TET1* transcript were also detected in other malignant cell lines: K562, Me45 and Raji (p<0.0001) compared to HCT 116 (Fig 3A). Both *TET1* mRNA and 5-hmdC levels have been abundant in HCT 116. Interestingly, we demonstrated that the level of 5-hmdC in DNA was positively correlated (r = 0.85, p = 0.0005) with the level of *TET1* transcript in all malignant cell lines used in this study (Fig 4).

A highest expression of *TET2* mRNA was noticed in HCT 116 cell line which was prominently higher than in the other investigated cell lines (Fig 3B).

The levels of *TET3* and *SMUG1* transcripts differed significantly between cell lines. HCT 116 cells were the most efficient in expressing *TET3* (p<0.0001) and *SMUG1* (p<0.0001) mRNA, whereas the other studied cell lines expressed both genes at very low levels (Fig 3C and 3D).

We also found the lowest level of *TDG* transcript in NHDF-Neo cells. A statistically significant higher level of *TDG* transcript was observed in all studied malignant cell lines, with the highest level in HCT 116 (Fig 3E).

## Discussion

Although many previous studies have centered around the measurement of 5-hmdC level, only a few have analyzed concentrations of 5-fdC, 5-cadC and 5-hmdU in various tissues [3, 10, 11]. The very low abundance of the latter modifications in the mammalian genome (approximately 3–4 orders of magnitude lower than that of 5-hmdC) makes accurate determination of their levels challenging.

Recent data clearly demonstrate that the huge inter-tissues differences in 5-hmdC level reflect mostly the cell proliferation status [12, 13]. However, it is not known whether this relationship applies to cells in culture. To provide a better insight into this issue, here we analyzed 5-hmdC and other modifications using a 2D-UPLC-MS/MS method with isotopically-labelled internal standards, a technique which has the advantage of relatively short run times, as well as the possibility of obtaining exceptionally high sensitivity and selectivity without compromising quality and validation criteria [6]. Furthermore, fully automated two-dimensional separation is an extremely useful technique for the analyses of biological samples by mass spectrometry-based methods. Using this method, we were able to detect all epigenetic DNA modifications in the cell types studied.

Using immune dot-blots Nestor et al. found substantial reduction in 5-hmdC level as cells from normal tissue adapt to cell culture [4]. In good agreement, we reported a 15-fold reduction of 5-hmdC level when compared CRC tissues with cultured colon cancer cells HCT 116. Similar, however milder changes have been found when K562 cell line was compared to the leukocytes of AML patients (Fig 2A). We extended data of Nestor et al. by demonstrating that the end product of 5-mdC oxidation, namely 5-cadC, was reduced in HCT 116 and K562 cell lines as compared to their primary tissues 16- and 40-fold, respectively (Fig 2C).

Strikingly, no differences were found between CRC tissues and HCT 116 cells for another epigenetic mark/TETs product, 5-fdC (Fig 2B) (see also below).

We also found substantial differences in 5-hmdC level among different cultured cell lines, with the highest level in normal fibroblasts (NHDF-Neo) and levels in malignant cell lines which decreased in the order HCT 116 > K562 > Me45 > Raji. The level of 5-hmdC inversely reflected to the level of expression of the *TET1* gene in the case of NHDF-Neo and HCT 116. Furthermore, we observed correlation between level of 5-hmdC and *TET1* transcript in all malignant cell lines (Fig 4).

Interestingly, recently published work suggests that *TET1* is primary responsible for 5-hmCyt formation, while *TET2* may be the main enzyme involved in 5-hmdC to 5-cadC conversion [14]. Among malignant cell lines, the level of 5-cadC was the highest in HCT 116 and Me45 (Fig 1D), where the highest expression of *TET2* was observed (Fig 3B). Moreover, the differences in the level of 5-cadC were not statistically significant because the levels (several modifications per 10^9^ deoxynucleosides) were on the border line of the detection limit. The levels of 5-fdC in different cell lines resembled those of 5-hmdC (Fig 1B and 1C).

Contrary to 5-hmdC, the level of 5-fdC was almost exactly the same in HCT 116 cells as in CRC tissues. However, in the case of K562 cell lines and their primary tissue (AML) level of 5-hmdC and 5-fdC was respectively 5- and 4.5-fold higher in the primary tissue.

As mentioned above, 5-hmdC is a relatively stable modification, and substantial differences in its level were shown to depend mostly on cell proliferation rate, with a linear negative correlation between the proliferation rate and the global level of 5-hmdC in several murine tissues [12]. Using similar tissues (brain, kidney, lung, heart, liver, muscle, spleen, gut, and thymus) we have documented a similar relationship for other mammalian species, pigs and rats [13], suggesting that this rule is likely to apply to all mammalian tissues and cell types. In that report we also found that the levels of 5-fdC and 5-hmdU were approximately three orders of magnitude lower than the levels of 5-hmdC in most tissues studied. Moreover, we found a considerable (20-fold) difference in 5-hmdC levels between non-proliferating cells (brain) and the fastest proliferating cells (thymus). However, the differences in 5-fdC and 5-cadC levels in the same tissues were markedly less prominent: four- and two-fold, respectively [13].

Here we found that in the cultured cell lines studied there were approximately two orders of magnitude differences between the 5-hmdC and 5-fdC levels, which was ten times less than in the mammalian tissues studied. Moreover, we did not observe any correlation between the proliferation rates of the malignant cells (reflected in their doubling times, Table 1) and the level of 5-hmdC or 5-fdC (Fig 1B and 1C).

**Table 1:** Doubling time of cells.

Cell line	Doubling time (h)
NHDF	25
HCT 116	8
K562	10
Me45	16
Raji	13
BL	11

However, normal cells (fibroblasts) which had the lowest proliferation rate (doubling time 25 h) had the highest level of 5-hmdC and 5-fdC, in agreement with Bachman et al [12]. Interestingly, in fibroblasts the expression levels of *TET1* were the lowest (Fig 3A). Together, these findings suggest that in cultured cells different factors are responsible for shaping the level of epigenetic DNA modifications than in mammalian tissues; while in tissues the cell proliferation rate is the main factor, in malignant cell lines *TET1* expression is directly linked to the level of epigenetic marks.

These findings raise an important question: what is the molecular background of these differences between normal tissues and cultured cells? While the involvement of TETs in all the modifications analyzed here raises no controversies, still little is known about the regulation of this process; specifically, it is unclear whether the level of the main substrate for TETs, namely 5-mdC, influences the formation of the products of iterative oxidation. It is also unclear why oxidation of 5-mdC either stops at 5-hmdC or proceeds to 5-fdC and 5-cadC. One potential explanation is the different affinity of TETs for 5-mdC, 5-hmdC and 5-fdC (for review see [15]). It is also possible that different proteins/factors recognize the modifications and determine their fate [16]. Recent evidence suggests that TET2 may yield 5-fdC and 5-cadC without the release and dilution of 5-hmdC, and it was postulated that consecutive steps of iterative oxidation are regulated by co-substrate levels [17].

Our study demonstrates that the level of 5-mdC (TET’s substrate), in cell culture, does not influence the level of 5-hmdC, the product of TET’s reaction, since the 5-mdC level in K562 cells is about two-fold lower than that in Me45 cells, while the 5-hmdC level is significantly higher (Fig 1A and 1B). We did not found substantial differences in the level of 5-hmdU, another product of TETs, among the cell lines studied with one exception; in HCT 116 cells this level was several fold lower than in the other cell types.

Recent evidence suggests that SMUG1 and TDG are the main enzymes involved in removal of 5-hmdU from DNA, and therefore their activity/expression (as a part of the BER pathway) may contribute to the level of this modified base in DNA. Since the *SMUG1* and *TDG* genes have a substantially higher expression in HCT 116 cells than in the other cell types (Fig 3D and 3E) this may explain the lowest level of 5-hmdU in these cells.

Of all the cell lines studied the lowest level of 5-hmdC was observed in Raji cells, which are human Burkitt lymphoma cells latently infected with Epstein-Barr virus (EBV). Raji cells have a statistically lower level of 5-mdC than their counterpart—EBV-negative cells (BL). A more prominent difference between these two cell types was observed for 5-hmdC, with a level about three-fold higher in EBV-negative in comparison with the EBV-positive cells (Fig 1B). These differences may be directly linked with the major epigenetic alteration upon EBV infection which was found in DNA of EBV-infected cells (reviewed in [18]).

In conclusion, this work shows a global erasure of 5-hmdC in DNA of cultured cells compared to DNA from different tissues, in agreement with Nestor et al. [4]. This is linked, at least partially, with a concomitant loss of TET1 activity. Moreover, comparison of cell line to primary cancer tissue (HCT 116 to CRC tissues and K562 to the leukocytes of AML patients) showed that, concerning 5-hmdC (first product of 5-mdC oxidative modification) and mainly 5-cadC (the end product of TETs) malignant cultured cell lines more closely resembled each other than the tissues from which they were derived. Furthermore, our work demonstrated that another TETs products 5-fdC and 5-hmdU doesn’t follow the same rule.

Our study shows, for the first time, that different cell lines in culture have different epigenetic profiles. Moreover, different types of malignant cells display characteristic profiles of DNA epigenetic marks which differ significantly. Recent work [19, 20] questions the use of cell lines as faithful models to study epigenetic processes linked to cancer development. However, our results suggest that analyses of a broader spectrum of epigenetic modifications, not restricted to 5-mdC, may lead to a better understanding of the mechanism(s) responsible for the formation of different cancer types.

## Supporting information

S1 Table Sequences of primers for RT-qPCR. (DOCX)
