# Correction: Profiles of a broad spectrum of epigenetic DNA modifications in normal and malignant human cell lines: Proliferation rate is not the major factor responsible for the 5-hydroxymethyl-2′-deoxycytidine level in cultured cancerous cell lines

**DOI:** 10.1371/journal.pone.0195819

**Published:** 2018-04-09

**Authors:** Marek Foksinski, Ewelina Zarakowska, Daniel Gackowski, Magdalena Skonieczna, Karolina Gajda, Dorota Hudy, Anna Szpila, Karol Bialkowski, Marta Starczak, Anna Labejszo, Jaroslaw Czyz, Joanna Rzeszowska-Wolny, Ryszard Olinski

The second grant number from the funder the Polish National Science Center is missing. The second grant number is: DEC-2015/19/B/NZ5/02208.
