# Supplementary material for: Profiles of a broad spectrum of epigenetic DNA modifications in normal and malignant human cell lines: Proliferation rate is not the major factor responsible for the 5-hydroxymethyl-2′-deoxycytidine level in cultured cancerous cell lines
Source: PLoS One. 2017 Nov 30;12(11):e0188856. doi: 10.1371/journal.pone.0188856 (PMC5708640; doi:10.1371/journal.pone.0188856)
Supplement: S1 Table — (DOCX) [file pone.0188856.s001.docx]

**S1 Table.** **Sequences of primers for RT-qPCR.**

| **Gene** | **Forward primer** | **Reverse primer** | **Tm^a^** |
| --- | --- | --- | --- |
| *TET1* | CCCCATCAAAGTCAGAGAAGG | GGTGGCTCTCCCTGTAAGTT | 56.8 |
| *TET2* | CCAGATTGTGTTTCCATTGC | GGCATTATCAGCATCATCAGC | 56.8 |
| *TET3* | CCGTGAGATGAGTCGTGAGG | GTGGTTTCATACCATGCCGC | 60.0 |
| *TDG* | CAAAACAACCAGTGGAACCC | GCTTCTGAAACACCATTAAAACG | 60.0 |
| *SMUG1* | GGCATCATCTACAATCCCGTG | GCCAAAAGGTCCAGGGTTCA | 60.0 |
| *GAPDH* | TTTGGCTACAGCAACAGGGTG | TTCCTCTTGTGCTCTTGCTGG | 56.8 and 60.0 |

^a^ melting temperature (°C)
